# Ever-Use and Curiosity About Cigarettes, Cigars, Smokeless Tobacco, and Electronic Cigarettes Among US Middle and High School Students, 2012–2014

**DOI:** 10.5888/pcd13.160151

**Published:** 2016-09-22

**Authors:** Alexander Persoskie, Elisabeth A. Donaldson, Brian A. King

**Affiliations:** Author Affiliations: Elisabeth A. Donaldson, Office of Science, Center for Tobacco Products, Food and Drug Administration, Silver Spring, Maryland; Brian A. King, Office on Smoking and Health, National Center for Chronic Disease Prevention and Health Promotion, Centers for Disease Control and Prevention, Atlanta, Georgia.

## Abstract

**Introduction:**

Among young people, curiosity about tobacco products is a primary reason for tobacco experimentation and is a risk factor for future use. We examined whether curiosity about and ever-use of tobacco products among US middle and high school students changed from 2012 to 2014.

**Methods:**

Data came from the 2012 and 2014 National Youth Tobacco Surveys, nationally representative surveys of US students in grades 6 through 12. For cigarettes, cigars, smokeless tobacco, and e-cigarettes (2014 only), students were classified as ever-users or never-users of each product. Among never-users, curiosity about using each product was assessed by asking participants if they had “definitely,” “probably,” “probably not,” or “definitely not” been curious about using the product.

**Results:**

From 2012 to 2014, there were declines in ever-use of cigarettes (26% to 22%; *P* = .005) and cigars (21% to 18%; *P* = .003) overall and among students who were Hispanic (cigarettes, *P* = .001; cigars, *P* = .001) or black (cigarettes, *P* = .004; cigars, *P* = .01). The proportion of never-users reporting they were “definitely not” curious increased for cigarettes (51% to 54%; *P* = .01) and cigars (60% to 63%; *P* = .03). Ever-use and curiosity about smokeless tobacco did not change significantly from 2012 to 2014. In 2014, the proportion of young people who were “definitely” or “probably” curious never-users of each product was as follows: cigarettes, 11.4%; e-cigarettes, 10.8%; cigars, 10.3%; and smokeless tobacco, 4.4%.

**Conclusion:**

The proportion of US students who are never users and are not curious about cigarettes and cigars increased. However, many young people remain curious about tobacco products, including e-cigarettes. Understanding factors driving curiosity can inform tobacco use prevention for youth.

## Introduction

Tobacco use is the leading preventable cause of death in the United States (1). Most tobacco use begins during youth or young adulthood, making this a critical period for preventing tobacco-related disease (2). To protect public health, the Family Smoking Prevention and Tobacco Control Act of 2009 gave the Food and Drug Administration the authority to regulate the manufacture, marketing, and distribution of tobacco products in the United States. Since then, tobacco products increased in complexity as noncigarette products came on the market and were increasingly promoted (3,4). For example, promotional expenditures for electronic cigarettes (e-cigarettes) was minimal at the start of 2010, but they increased to $12 million in 2011, $22 million in 2012, and over $28 million in the second quarter of 2013 (4). Moreover, advertisements for these products are reaching adolescents and young adults: in 2014, 68.9% of middle and high school students (18.3 million) were exposed to e-cigarette advertisements through retail stores, the Internet, TV and movies, and newspapers and magazines (4–6). Additionally, research indicates high availability of little cigars and cigarillos, including sales of single sticks, low-priced products, and flavored products (7).

Reflecting this change in tobacco products, tobacco use patterns among US young people changed substantially in recent years (8). During 2011–2014, past 30-day use of e-cigarettes and hookah increased considerably among high school students, thus offsetting declines in cigarette and cigar smoking. These shifts in product use, coupled with no change in smokeless tobacco use, resulted in no change in the overall prevalence of any tobacco use (24.2% vs 24.6%) among high school students during this period (8). In 2013, 46% of all US high school students and 18% of middle-school students reported having used a tobacco product (9).

Ever-use of tobacco products among young people is important from a public health perspective for several reasons. Product trial is a critical step in the product adoption process (10), and tobacco companies have invested considerably in promotional activities to encourage product trial and experimentation (11–14). The addictive effects of nicotine can develop after intermittent use of cigarettes by young people (15), and early age of first use of cigarettes is associated with greater nicotine dependence and heavier smoking in adulthood (2). Furthermore, nicotine may adversely affect the developing adolescent brain (1,16).

One potential driver of product trial and shifting patterns of tobacco use is curiosity about tobacco products. In studies that ask tobacco users their reasons for initial tobacco experimentation, curiosity is one of the most commonly cited reasons (17–20). Moreover, longitudinal research among young people found that curiosity about cigarettes is an important risk factor for smoking in the future (21,22). One study of young people who never smoked found that 49% of those who reported “definitely” or “probably” being curious about cigarettes went on to experiment with cigarettes within the next 3 years, whereas only 21% of those who were “definitely not” curious went on to experiment with tobacco (21). Even after accounting for a measure of young people’s susceptibility to cigarette smoking (ie, young people who report they would experiment with cigarettes or smoke a cigarette if offered one by a friend), curiosity was found to be useful for identifying which young people who had never smoked would progress to experimentation and established smoking (22).

Researchers of tobacco curiosity theorized that curiosity “indicates interest, even in the absence of intentions to use” a product (23). Tobacco curiosity is thought to be generated in part by exposure to tobacco advertising and peer use (21,23). Antitobacco communications and warnings about the effects of tobacco use would be expected to reduce curiosity or to deter young people from acting on their curiosity; however, only 1 known study assessed this hypothesis, finding no significant association between self-reported exposure to on-pack warning labels for cigarettes and smokeless tobacco and curiosity about these products (23).

Little is known about how young people’s curiosity is changing as US tobacco products change. Given the longitudinal research indicating that curiosity about smoking predisposes young people to future experimentation and use of cigarettes, changes in young people’s curiosity are important to monitor as they may presage changes in use. One analysis of the 2012 National Youth Tobacco Survey (NYTS) found that 13.4% of young people who had never used tobacco were “definitely” or “probably” curious about cigarettes, with 9.6% curious about cigars and 4.1% about smokeless tobacco (23). No data on more recent trends in young people’s curiosity about these products are reported. To address gaps in the scientific literature, our analysis assessed whether young people’s curiosity about cigarettes, cigars, and smokeless tobacco changed between 2012 and 2014. Also, we assessed young people’s curiosity about e‑cigarettes in 2014, the first year for which national data on young people’s curiosity about e-cigarettes were available.

## Methods

Data used were from NYTS, a cross-sectional, school-based, self-administered, pencil-and-paper survey of US middle and high school students. A 3-stage cluster sampling procedure was used to generate a nationally representative sample of students attending public or private schools (grades 6–12). This report includes data from the 2012 (n = 24,658) and 2014 (n = 22,007) waves; response rates were 73.6% and 73.3%, respectively. The NYTS research protocol was approved by the US Office of Management and Budget; no ethics approval was required for this analysis because only secondary, de‑identified data were used.

### Measures

Ever-use of each tobacco product was assessed by asking participants if they had ever tried cigarette smoking, even 1 or 2 puffs; had ever tried smoking cigars, cigarillos, or little cigars, even 1 or 2 puffs; had ever used chewing tobacco, snuff, or dip, even just a small amount; and had ever tried an electronic cigarette. Students who responded yes were considered “ever-users” and those who responded no were considered “never-users” of the product in question.

Never-users’ curiosity about each product (cigarettes, cigars, smokeless tobacco, e‑cigarettes) was assessed. Curiosity about cigarette, cigar, and smokeless tobacco were assessed in 2012 and 2014 by asking participants if they had ever been curious about smoking a cigarette; smoking a cigar, cigarillo, or little cigar; and using chewing tobacco, snuff, or dip. E-cigarette curiosity was assessed in 2014 by asking participants if they had ever been curious about using an electronic cigarette or e-cigarette. For all items, response options were, “definitely yes,” “probably yes,” “probably not,” and “definitely not.”

Consistent with prior research (21–23), responses of “definitely yes” and “probably yes” were analyzed as a single category. We did so because prior longitudinal studies validating this measure of curiosity found that the likelihood of experimentation and use was lowest for participants who responded “definitely not,” increased for participants who responded “probably not,” and was highest for those who responded either “probably yes” or “definitely yes” (21,22).

Assessed sociodemographics were sex (male, female), race/ethnicity (non-Hispanic white, non-Hispanic black, Hispanic, and other), and age (9–14, 15–16, ≥17 y).

### Analysis

For each product, students were classified as ever-users or never-users who were “definitely not” curious, “probably not” curious, “probably” curious, or “definitely” curious. By using Stata 14 (StataCorp LP), weighted point estimates and 95% confidence intervals for curiosity and ever-use were calculated for cigarettes, cigars, and smokeless tobacco in 2012 and 2014, and for e-cigarettes in 2014. We used χ^2^ tests to assess changes in ever-use and curiosity from 2012 to 2014 for cigarettes, cigars, and smokeless tobacco. When significant differences emerged, follow-up χ^2^ tests examined whether the proportion in each group (eg, never-users who were “definitely not” curious) changed from 2012 to 2014. The threshold for significance was a *P* value < .05.

## Results

In 2012 and 2014, the distribution of respondents by sex was similar: 48.9% of respondents in 2012 were female compared with 49.8% of respondents in 2014 (Table 1). The distribution of respondents by age was also similar in 2012 and 2014; nearly half of respondents were aged 9 to 14 (almost 49% in both years), while approximately 29% of respondents were aged 14 to 16, and 22% were aged 17 or older. Slightly more respondents were non-Hispanic blacks in 2014 than in 2012 (14.6% in 2014 and 13.9% in 2012); however, this difference was not significant.

**Table 1:** Demographic Characteristics of Participants, National Youth Tobacco Survey, United States, 2012 and 2014

Characteristic	N (Weighted %)	
	2012	2014
**Overall**	24,658	22,007
**Sex**		
Female	12,275 (48.9)	10,645 (49.8)
Male	12,369 (51.1)	11,150 (50.2)
**Age, y**		
9–14	12,627 (48.5)	11,296 (48.7)
15–16	6,430 (29.2)	5,839 (29.0)
≥17	5,498 (22.3)	4,715 (22.3)
**Race/ethnicity **		
White, non-Hispanic	11,814 (53.9)	8,820 (53.2)
Black, non-Hispanic	3,114 (13.9)	3,226 (14.6)
Hispanic	5,733 (21.7)	6,081 (21.9)
Other	3,211 (10.6)	2,673 (10.3)

From 2012 to 2014, the proportion of students who were never users and “definitely not” curious about cigarettes increased significantly overall (51.2% to 54.3%). Significant increases also occurred among students who were male (49.6% to 54.0%), aged 17 or older (39.7% to 45.0%), non-Hispanic black (53.5% to 58.5%), or Hispanic (45.5% to 49.4%). In contrast, the proportion of students who were never-users of cigarettes and “probably” or “definitely” curious about cigarettes did not change significantly (10.8% in 2012 and 11.4% in 2014). Similarly, no significant change was observed across demographic groups with the exception of Hispanics, for whom “probably” or “definitely” curious increased from 2012 (12.2%) to 2014 (14.1%) (*P* = .03). The proportion of students who were never-users and “probably not” curious about cigarettes did not change significantly from 2012 to 2014, either overall (11.6% in 2012 and 11.9% in 2014) or within any of the assessed demographic groups (Table 2).

**Table 2:** Weighted Prevalence of Ever-Use and Level of Curiosity About Cigarettes and Cigars by Year and Demographic Characteristics, National Youth Tobacco Survey, United States, 2012 and 2014

Demographic Category/Year (n)	Cigarettes				Cigars			
	Ever User, % (95% CI)	“Definitely” or “Probably” Curious Never-User, % (95% CI)	“Probably Not” Curious Never-User, % (95% CI)	“Definitely Not” Curious Never-User, % (95% CI)	Ever User, % (95% CI)	“Definitely” or “Probably” Curious Never-User, % (95% CI)	“Probably Not” Curious Never-User, % (95% CI)	“Definitely Not” Curious Never-User, % (95% CI)
**Overall**								
2012 (24,658)	26.4^a^^,^ b (24.3–28.6)	10.8^a^ (10.2–11.4)	11.6^a^ (10.9–12.3)	51.2^a^^,^ b (49.5–52.9)	21.2^a^^,^ b (19.5–23.0)	9.9^a^ (9.2–10.6)	8.8^a^ (8.2–9.4)	60.1^a^^,^ b (58.5–61.8)
2014 (22,007)	22.4^a^^,^ b (20.8–24.1)	11.4^a^ (10.8–12.1)	11.9^a^ (11.2–12.6)	54.3^a^^,^ b (52.8–55.8)	17.6^a^^,^ b (16.2–19.2)	10.3^a^ (9.6–11.0)	9.3^a^ (8.7–9.9)	62.8^a^^,^ b (61.0–64.5)
**Sex**								
**Female**								
2012 (12,275)	24.4^a^^,^ b (22.1–26.8)	11.7^a^ (10.9–12.5)	11.0^a^ (10.1–11.9)	52.9^a^ (50.9–54.9)	17.1^a^^,^ b (15.3–19.1)	8.6^a^ (7.9–9.4)	8.0^a^ (7.4–8.8)	66.2^a^ (64.1–68.2)
2014 (10,645)	21.1^a^^,^ b (19.5–22.9)	12.3^a^ (11.3–13.2)	12.0^a^ (11.3–12.8)	54.6^a^ (52.9–56.4)	14.2^a^^,^ b (12.9–15.7)	9.3^a^ (8.5–10.1)	9.1^a^ (8.4–9.8)	67.4^a^ (65.5–69.2)
**Male**								
2012 (12,369)	28.3^a^^,^ b (26.2–30.5)	9.9^a^ (9.2–10.7)	12.2^a^ (11.4–13.0)	49.6^a^^,^ b (47.8–51.4)	25.2^a^^,^ b (23.3–27.2)	11.1^a^ (10.2–12.0)	9.5^a^ (8.7–10.4)	54.2^a^^,^ b (52.3–56.0)
2014 (11,150)	23.6^a^^,^ b (21.8–25.5)	10.6^a^ (10.0–11.3)	11.7^a^ (10.7–12.8)	54.0^a^^,^ b (52.3–55.8)	21.0^a^^,^ b (19.1–23.0)	11.4^a^ (10.5–12.4)	9.4^a^ (8.7–10.2)	58.2^a^^,^ b (56.0–60.3)
**Age, y**								
**9–14**								
2012 (12,627)	14.3^a^^,^ b (12.8–15.9)	12.2^a^ (11.4–13.0)	13.6^a^ (12.8–14.5)	59.9^a^ (58.2–61.6)	8.4 (7.5–9.5)	10.0 (9.0–11.1)	9.6 (8.8–10.5)	72.0 (70.6–73.4)
2014 (11,296)	11.8^a^^,^ b (10.3–13.5)	12.5^a^ (11.6–13.5)	13.9^a^ (13.2–14.6)	61.8^a^ (60.2–63.5)	6.9 (6.1–7.8)	9.4 (8.5–10.3)	9.5 (8.8–10.3)	74.2 (72.5–75.8)
**15–16**								
2012 (6,430)	33.2 (30.0–36.5)	10.3 (9.2–11.4)	10.8 (9.8–11.9)	45.7 (42.9–48.5)	28.0^a^^,^ b (25.4–30.7)	10.1^a^^,^ b (9.1–11.1)	8.8^a^ (7.9–9.8)	53.1^a^ (50.3–55.9)
2014 (5,839)	28.6 (25.9–31.5)	10.7 (9.6–11.8)	11.7 (10.7–12.8)	49.0 (46.7–51.3)	22.8^a^^,^ b (20.8–24.9)	11.9^a^^,^ b (10.7–13.2)	10.2^a^ (9.2–11.4)	55.1^a^ (52.9–57.2)
**≥17**								
2012 (5,498)	43.6^a^^,^ b (40.6–46.7)	8.5^a^ (7.4–9.6)	8.2^a^ (7.1–9.4)	39.7^a^^,^ b (37.5–42.1)	40.2^a^^,^ b (37.7–42.7)	9.3^a^ (8.3–10.5)	7.0^a^ (5.9–8.2)	43.5^a^^,^ b (41.5–45.5)
2014 (4,715)	37.3^a^^,^ b (33.7–41.0)	10.0^a^ (9.0–11.1)	7.7^a^ (6.5–9.1)	45.0^a^^,^ b (41.5–48.5)	34.4^a^^,^ b (30.9–38.0)	10.3^a^ (9.3–11.4)	7.4^a^ (6.3–8.8)	47.9^a^^,^ b (44.4–51.4)
**Race/ethnicity**								
**Non-Hispanic white**								
2012 (11,814)	25.1 (22.6–27.9)	9.7 (8.9–10.6)	12.5 (11.6–13.6)	52.6 (50.5–54.7)	19.7 (17.9–21.7)	8.5 (7.9–9.1)	9.1 (8.4–9.8)	62.7 (60.7–64.6)
2014 (8,820)	21.6 (19.6–23.8)	10.2 (9.5–10.9)	12.7 (11.8–13.7)	55.5 (53.6–57.3)	17.0 (15.2–19.0)	9.1 (8.3–10.0)	8.9 (8.1–9.7)	64.9 (62.5–67.3)
**Non-Hispanic black**								
2012 (3,114)	27.1^a^^,^ b (23.3–31.3)	11.0^a^ (9.5–12.8)	8.4^a^ (7.3–9.6)	53.5^a^^,^ b (50.2–56.7)	27.8^a^^,^ b (23.7–32.4)	12.3^a^ (10.9–13.8)	6.2^a^^,^ b (5.0–7.6)	53.7^a^^,^ b (50.0–57.2)
2014 (3,226)	20.1^a^^,^ b (17.9–22.6)	12.2^a^ (10.9–13.5)	9.2^a^ (7.9–10.8)	58.5^a^^,^ b (56.1–60.8)	20.8^a^^,^ b (17.8–24.2)	11.8^a^ (10.2–13.6)	8.0^a^^,^ b (7.1–9.0)	59.3^a^^,^ b (56.1–62.5)
**Hispanic**								
2012 (5,733)	30.8^a^^,^ b (27.9–33.8)	12.2^a^^,^ b (11.1–13.4)	11.5^a^ (10.3–12.9)	45.5^a^^,^ b (42.9–48.1)	23.1^a^^,^ b (20.9–25.6)	11.7^a^ (10.5–13.0)	9.4^a^ (8.1–10.8)	55.8^a^ (53.4–58.2)
2014 (6,081)	24.9^a^^,^ b (22.6–27.3)	14.1^a^^,^ b (13.0–15.3)	11.6^a^ (10.6–12.7)	49.4^a^^,^ b (47.1–51.6)	18.1^a^^,^ b (16.4–20.0)	12.7^a^ (11.4–14.0)	10.6^a^ (9.6–11.6)	58.6^a^ (56.8–60.4)
**Other**								
2012 (3,211)	24.6 (21.5–27.8)	13.3 (11.5–15.3)	11.0 (9.6–12.6)	51.1 (48.4–53.9)	17.7 (15.0–20.7)	10.5 (9.2–12.0)	9.2 (8.1–10.5)	62.5 (59.6–65.3)
2014 (2,673)	23.7 (20.5–27.2)	12.9 (11.2–14.9)	12.4 (10.6–14.4)	51.0 (48.3–53.7)	16.0 (13.0–19.5)	11.1 (9.5–13.0)	10.5 (8.4–12.9)	62.5 (59.2–65.6)

a Indicates a significant difference in the overall distribution of ever-use and curiosity about a product between 2012 and 2014 (*P* < .05), estimated based on a χ^2^ test of association weighted for the survey design.

b Indicates a significant difference in the cell proportion between 2012 and 2014 (*P* < .05), estimated based on a follow-up χ^2^ test. Changes in cell proportion were tested only when the overall distribution of ever-use and curiosity changed significantly between survey years.

Overall, ever-use of cigarettes decreased from 2012 to 2014 (from 26.4% to 22.4%, *P* = .005; Table 2). Significant decreases in ever-use were also observed within specific subgroups: females (24.4% to 21.1%; *P* = .03), males (28.3% to 23.6%; *P* = .003), non-Hispanic black students (27.1% to 20.1%; *P* = .004), Hispanic students (30.8% to 24.9%; *P* = .001), students aged 9 to 14 (14.3% to 11.8%; *P* = .03), and students aged 17 or older (43.6% to 37.3%; *P* =.04) (Table 2).

The proportion of students who were never-users and “definitely not” curious about cigars significantly increased overall (60.1% to 62.8%), as well as among students who were male (54.2% to 58.2%), aged 17 or older (43.5% to 47.9%), or black (53.7% to 59.3%). In contrast, the proportion of students who were never-users of cigars and “probably” or “definitely” curious about cigars did not significantly change overall (9.9% in 2012 to 10.3% in 2014). Similarly, no significant change occurred within any demographic group with the exception of students aged 15 to 16 (10.1% to 11.9%; *P* = .03). The proportion of students who were never-users and “probably not” curious about cigars did not change significantly from 2012 to 2014 overall (8.8% to 9.3%), but did increase among non-Hispanic black students (6.2% to 8.0%; *P* =.03) (Table 2).

Overall, ever-use of cigars significantly decreased from 2012 to 2014 (from 21.2% to 17.6%; *P* = .003). Ever-use of cigars also decreased within specific groups, including females (17.1% to 14.2%; *P* = .01), males (25.2% to 21.0%; *P* = .005), non-Hispanic blacks (27.8% to 20.8%; *P* = .01), Hispanics (23.1% to 18.1%; *P* = .001), students aged 15 to 16 (28.0% to 22.8%; *P* = .003), and students aged 17 or older (40.2% to 34.4%; *P* = .009) (Table 2).

Ever-use and curiosity about smokeless tobacco did not change significantly between 2012 and 2014, either overall or within any of the assessed demographic groups (Table 3). Overall, in 2014, ever-use of smokeless tobacco was 8.3%, while 4.4% were never-users but “definitely” or “probably” curious about smokeless tobacco, 6.0% were never-users and “probably not” curious, and 81.3% were never-users and “definitely not” curious.

**Table 3:** Weighted Prevalence of Ever-Use and Level of Curiosity About Smokeless Tobacco and Electronic Cigarettes by Year and Demographic Characteristics, National Youth Tobacco Survey, United States, 2012 and 2014

Demographic Category/Year (n)	Smokeless Tobacco				Electronic Cigarettes			
	Ever User, % (95% CI)	“Definitely” or “Probably” Curious Never-User, % (95% CI)	“Probably Not” Curious Never-User, % (95% CI)	“Definitely Not” Curious Never-User, % (95% CI)	Ever User, % (95% CI)	“Definitely” or “Probably” Curious Never-User, % (95% CI)	“Probably Not” Curious Never-User, % (95% CI)	“Definitely Not” Curious Never-User, % (95% CI)
**Overall**								
2012 (24,658)	9.6 (8.3–11.1)	4.7 (4.2–5.2)	5.7 (5.2–6.2)	80.0 (78.6–81.4)	NA			
2014 (22,007)	8.3 (7.1–9.7)	4.4 (4.0–4.8)	6.0 (5.4–6.6)	81.3 (79.6–82.8)	19.8 (18.0–21.8)	10.8 (10.2–11.4)	10.0 (9.4–10.5)	59.4 (57.3–61.5)
**Sex**								
**Female**								
2012 (12,275)	4.4 (3.7–5.2)	3.8 (3.2–4.5)	4.7 (4.2–5.2)	87.1 (86.0–88.2)	NA			
2014 (10,645)	3.7 (3.0–4.5)	3.7 (3.3–4.2)	5.3 (4.7–6.0)	87.2 (85.9–88.4)	18.2 (16.4–20.2)	11.3 (10.4–12.3)	9.9 (9.2–10.7)	60.6 (58.2–62.9)
**Male**								
2012 (12,369)	14.7 (12.8–16.9)	5.6 (5.0–6.2)	6.6 (5.9–7.3)	73.1 (71.0–75.1)	NA			
2014 (11,150)	12.8 (10.9–15.0)	5.1 (4.6–5.7)	6.7 (6.0–7.5)	75.4 (73.1–77.5)	21.3 (19.3–23.6)	10.2 (9.5–11.0)	10.0 (9.3–10.8)	58.4 (56.0–60.7)
**Age, y**								
**9–14**								
2012 (12,627)	4.4 (3.7–5.4)	5.6 (4.7–6.6)	6.6 (5.8–7.4)	83.4 (81.8–84.8)	NA			
2014 (11,296)	4.0 (3.1–5.1)	5.0 (4.9–5.5)	6.8 (5.9–7.7)	84.2 (82.6–85.7)	10.8 (9.3–12.5)	11.1 (10.1–12.1)	10.7 (9.9–11.6)	67.4 (65.5–69.2)
**15–16**								
2012 (6,430)	12.0 (10.2–14.2)	4.2 (3.6–4.8)	5.5 (4.8–6.4)	78.3 (76.0–80.3)	NA			
2014 (5,839)	10.0 (8.4–11.8)	4.4 (3.7–5.2)	5.8 (5.0–6.6)	79.8 (77.4–82.1)	25.9 (23.1–28.9)	11.3 (10.4–12.3)	10.1 (9.1–11.0)	52.7 (49.4–55.9)
**≥17**								
2012 (5,498)	17.7 (15.4–20.3)	3.4 (2.8–4.0)	3.8 (3.2–4.6)	75.1 (72.7–77.3)	NA			
2014 (4,715)	15.3 (13.0–17.8)	3.3 (2.7–4.1)	4.6 (4.0–5.3)	76.8 (74.4–79.0)	31.4 (27.6–35.4)	9.4 (8.4–10.4)	8.3 (7.1–9.7)	50.9 (47.0–54.9)
**Race/ethnicity**								
**Non-Hispanic white**								
2012 (11,814)	12.2 (10.5–14.1)	4.4 (3.9–5.0)	5.7 (5.2–6.2)	77.7 (75.7–79.5)	NA			
2014 (8,820)	11.2 (9.6–13.0)	4.4 (3.8–5.0)	6.1 (5.4–6.8)	78.3 (76.1–80.3)	20.4 (18.1–23.0)	9.4 (8.6–10.2)	10.3 (9.5–11.0)	59.9 (57.1–62.6)
**Non-Hispanic black**								
2012 (3,114)	4.0 (3.0–5.4)	4.0 (3.0–5.2)	3.3 (2.4–4.4)	88.7 (86.9–90.4)	NA			
2014 (3,226)	2.5 (2.0–3.3)	3.3 (2.5–4.2)	3.6 (3.1–4.3)	90.6 (89.3–91.6)	14.3 (12.1–16.7)	12.1 (10.6–13.8)	7.6 (6.5–8.8)	66.0 (62.7–69.2)
**Hispanic**								
2012 (5,733)	7.5 (6.2–9.2)	5.5 (4.7–6.3)	6.7 (5.8–7.6)	80.3 (78.5–82.0)	NA			
2014 (6,081)	5.7 (4.7–6.8)	5.5 (4.8–6.2)	7.2 (5.9–8.7)	81.6 (79.7–83.3)	22.9 (20.5–25.2)	13.3 (12.2–14.5)	11.0 (10.0–12.1)	52.9 (50.6–55.1)
**Other**								
2012 (3,211)	8.3 (6.6–10.3)	5.3 (4.3–6.4)	6.3 (5.3–7.6)	80.1 (78.0–82.0)	NA			
2014 (2,673)	6.4 (5.0–8.2)	5.1 (3.7–6.9)	6.2 (5.3–7.3)	82.3 (80.0–84.3)	19.6 (16.5–23.1)	12.5 (11.0–14.3)	10.1 (8.9–11.3)	57.8 (54.6–61.0)

Abbreviation: NA, not assessed.

In 2014, 19.8% of students had ever-used e-cigarettes (95% CI, 18.0–21.8), 10.8% were never-users but were “definitely” or “probably” curious about e-cigarettes, and 10.0% were never-users and “probably not” curious about e-cigarettes.

## Discussion

This analysis revealed that the proportion of students who were never-users and “definitely not” curious about cigarettes and cigars increased from 2012 to 2014. These changes were observed overall and within specific subgroups, including groups that had high rates of ever-use of cigarettes and cigars in 2012 (students who were non-Hispanic black, Hispanic, or aged 17 or older). However, for some subgroups, no change occurred in patterns of ever-use and curiosity about cigarettes and cigars across years, including for students who were non-Hispanic white or from “other races/ethnicities.” Moreover, the distribution of ever-use and curiosity about smokeless tobacco did not significantly change overall or for any demographic subgroup assessed. Finally, levels of ever-use and curiosity about e-cigarettes — assessed for the first time in 2014 — approached the levels observed for traditional cigarettes and cigars. These findings underscore the importance of addressing the factors driving curiosity about tobacco, which can inform ongoing and future efforts to prevent all forms of tobacco use by US students.

In general, increases were not observed in the proportion of students who were never-users and curious about products. However, there was an increase in the proportion of Hispanic students who were never-users and curious about cigarettes, and there was an increase in the proportion of students aged 15 to 16 years who were never-users and curious about cigars. In these groups, the increases in proportions of never-users who were curious may reflect progress in preventing young people from using tobacco, because these changes were accompanied by decreases in ever-use of cigarettes and cigars. Such a pattern (ie, a reduction in ever-use and an increase in the proportion of students who were never-users but curious) would be expected if some types of tobacco control efforts (eg, increasing cigarette excise taxes) prevented ever-use by students but did not affect their curiosity. Thus, some students would remain curious never-users rather than progressing to ever-users, which would benefit public health and prevent young people from using tobacco.

Over 2 years, the lack of significant changes in ever-use and curiosity about cigarettes and cigars among certain subgroups (eg, students who were white or from “other races/ethnicities”) may not seem noteworthy. These constructs are measured over the lifetime, and tobacco control initiatives implemented after students have already experimented with a tobacco product will not change their status as an ever-user, although such initiatives may affect their current tobacco use. Therefore, it is noteworthy that we observed significant decreases in the proportions of Hispanic and black students who were ever-users of cigarettes and cigars and significant increases in the proportions of these students who were noncurious never-users of these products. We are uncertain about the extent to which these changes reflect shifts in the implementation of tobacco control measures, such as comprehensive smoke-free air laws and tobacco taxes (24,25).

Ever-use and curiosity about smokeless tobacco products did not change significantly, but there was a nonsignificant trend for all subgroups in ever-use being lower in 2014 than in 2012. Continuing to monitor trends is important, given the known adverse health consequences of smokeless tobacco use, recent media discussion of the risks of smokeless tobacco products compared with cigarettes (26), and changing patterns of marketing and advertising of these products (27,28).

Levels of ever-use and curiosity about e-cigarettes, which were assessed for the first time in 2014, approached the levels observed for traditional cigarettes and cigars. This is consistent with data documenting rapid increases in the use of these products by US students: in 2014, e-cigarettes were the most commonly used tobacco product by middle (3.9%) and high (13.4%) school students (8). Use of tobacco in any form, whether it be combusted, noncombusted, or electronic, is unsafe (1). Regardless of mode of exposure to nicotine (inhaling, chewing, or electronic), such exposure during adolescence (a critical time for brain development) may have lasting adverse consequences for brain development. Nicotine exposure during adolescence also causes addiction and may lead to sustained use of tobacco products (1). Accordingly, curiosity about emerging tobacco products among students, coupled with the rapid changes in the use of these products by young people, underscores the importance of sustained strategies to prevent and reduce the use of all tobacco products among US youth, including emerging products such as e-cigarettes.

Strengths of this analysis include the large sample size that allowed for robust estimates of ever-use and curiosity within subgroups of adolescents varying by sex, age, and race/ethnicity. Additionally, we used a measure of curiosity that longitudinally predicts cigarette smoking and is associated in theoretically expected ways with exposure to advertising for other tobacco products (eg, cigars, smokeless tobacco) (23). Nonetheless, the curiosity measure was a self-reported measure that is subject to social desirability and recall bias. Our analysis was also limited in its assessment of tobacco products. It focused on the subset of products for which the NYTS assessed curiosity in either 2012 or 2014, which did not include other types of tobacco products such as hookah (9). Additionally, diverse products (cigars, cigarillos, and little cigars) were grouped into single categories. Asking separately about each product subtype may reveal meaningful differences across subtypes.

Although progress was made in reducing middle- and high-school students’ ever-use and curiosity about some tobacco products, continued efforts are critical to prevent them from using any tobacco products. The finding that levels of curiosity differed by product type and that curiosity changed over time raises questions about factors that affect curiosity about tobacco. Potential influences to investigate in future research include exposure to tobacco advertising (eg, on social media) (5,29,30), interest in flavored products (31), exposure to tobacco use in public places (32), and social influences from, for example, peers or family. Investigating the effects of tobacco control policies and tobacco prevention messages on young people’s curiosity about tobacco products may allow for improved design and targeting of these strategies and messages. Taken as a whole, these results highlight the value and feasibility of sustained efforts to monitor young people’s ever-use of and curiosity about tobacco products and to implement strategies that focus on the diversity of tobacco products to prevent tobacco use by adolescents.
